# Design and performance analysis of 3D-printed stiffness gradient femoral scaffold

**DOI:** 10.1186/s13018-023-03612-z

**Published:** 2023-02-18

**Authors:** Linlin Liu, Chang Liu, Congying Deng, Xin Wang, Xiangde Liu, Maolin Luo, Shuxian Wang, Juncai Liu

**Affiliations:** 1 School of Advanced Manufacturing Engineering, Chongqing University of Posts and Telecommunications, Chongqing, 400065 China; 2 Department of Orthopaedics, The Affiliated Hospital of Southwest Medical University, Sichuan Provincial Laboratory of Orthopaedic Engineering, Luzhou, 646000 Sichuan China

**Keywords:** Femur defect repair, Stiffness gradient scaffold, Integrated fixation, Finite element simulation

## Abstract

Studies on 3D-printed porous bone scaffolds mostly focus on materials or structural parameters, while the repair of large femoral defects needs to select appropriate structural parameters according to the needs of different parts. In this paper, a kind of stiffness gradient scaffold design idea is proposed. Different structures are selected according to the different functions of different parts of the scaffold. At the same time, an integrated fixation device is designed to fix the scaffold. Finite element method was used to analyze the stress and strain of homogeneous scaffolds and the stiffness gradient scaffolds, and the relative displacement and stress between stiffness gradient scaffolds and bone in the case of integrated fixation and steel plate fixation. The results showed that the stress distribution of the stiffness gradient scaffolds was more uniform, and the strain of host bone tissue was changed greatly, which was beneficial to the growth of bone tissue. The integrated fixation method is more stable, less stress and evenly distributed. Therefore, the integrated fixation device combined with the design of stiffness gradient can repair the large femoral bone defect well.

## Introduction

Repair of large bone defects caused by bone tumors is one of the most difficult problems for surgeons. Femoral tumors often occur in young and middle-aged people, and amputation will seriously affect the quality of life of patients. With the progress of early diagnosis, surgical techniques and design and manufacture of femoral prosthesis, limb salvage surgery has become the best choice for the treatment of femoral malignant tumor [1]. 3D-printed porous titanium alloy bone scaffolds have been applied more and more clinically due to their good mechanical and biological properties [2]. Porous titanium alloy can avoid the stress shielding effect caused by the mismatch between the stiffness of the scaffold and the bone tissue, and allow the bone tissue to grow into the inner part of the scaffold to form long-term biological fixation, which is a good bone defect repair material [3].

Aseptic loosening and prosthesis fracture are the two most common conditions of femoral prosthesis failure [4]. Aseptic loosening is mainly caused by the failure of bone tissue to grow into the scaffold. At present, researchers focus on how to change the shape and pore size of the porous structure to make better bone tissue ingrowth [5, 6]. The research results of Pires et al. [7] showed that the triply periodic minimal surfaces were a good bone repair structure. Zhang et al. [8] analyzed the mechanical and biological properties of regular porous scaffolds, and the results showed that 3D-printed porous titanium alloy scaffolds are good repair materials for load-bearing bone tissues. Wang et al. [9] designed an irregular porous scaffold, and the results showed that the scaffold had good mechanical and biological properties and could be used for repairing large bone defects. Chantarapanich et al. [10] set up a scaffold library and obtained a scaffold with good mechanical properties by using the composite scaffold structure established by different scaffold structures. However, these studies only focused on the mechanical properties of the bone scaffold structure itself and did not consider the effect of mechanical stimulation on the growth of bone tissue. Mechanical stimulation can promote the growth of bone tissue to a certain limit. There is a direct relationship between the amount of bone production in porous titanium alloy scaffolds and the strain of corresponding parts [11, 12]. Studies have shown that bone strain in the range of 1–5% is conducive to the formation of bone tissue [13]. Mehta et al. [14] showed that the strain of about 1% in the early stage of bone healing is conducive to the growth of bone tissue. For the repair of load-bearing bone defects, the traditional method is to use porous scaffolds whose mechanical properties match the bone tissue for repair [15]. To achieve the strain window described above, scaffolds that are softer than bone tissue should be used at the site of bone defect. However, the repair of load-bearing bone defects requires scaffolds with sufficient mechanical strength to play a mechanical supporting role. Moreover, human bone tissue is a hierarchical structure, and the mechanical and biological properties of cortical bone and cancellous bone are very different [16]. If the same structure is used to repair bone tissue in different parts, it will not have good repair effect. Gradient structure scaffolds provide a new design scheme for bone defect scaffolds, which can design different topological structures or porosity to meet the mechanical requirements of different parts [17]. Han et al. [18] designed a porous gradient scaffold with controllable porosity and mechanical properties, demonstrating the advantages of porous gradient scaffolds in the field of bone repair. The study of Caparros et al. [19] showed that gradient porous scaffolds were more suitable for cell proliferation than homogeneous porous scaffolds. The research results of Kumar et al. [20] show that porous gradient scaffolds can provide the optimal combination of porosity and mechanical properties for bone defects. However, these studies only focused on the porous gradient scaffold itself, and no targeted porous gradient scaffold design was carried out for specific bone defect sites.

Prosthesis fractures are caused by the improper fixation of the prosthesis. In traditional fixation, steel plate and fixation screw are used to fix the prosthesis. In this way, the steel plate cannot completely fit the bone tissue, which is easy to cause stress concentration and fatigue injury [21, 22]. To better fix the bone graft, researchers have proposed different fixation methods. Zhao et al. [23] designed a method of cross-fixation on porous scaffolds to fix the prosthesis, but this method is complicated and requires a high level of expertise during surgery. Lu et al. [24] fixed the scaffold for repairing large femoral bone defects in different directions by combining 3D printing and steel plate fixation, but such fixation increased the number of screws, which was not conducive to the later recovery of patients. 3D printing technology allows designers to design personalized femoral scaffold fixation devices, which are integrated with the porous scaffold through 3D printing, avoiding displacement between the device and the stent and simplifying the surgical process.

Different from the porous structure designed with stiffness gradient, this paper designed a stiffness gradient scaffold for large segmental bone defects and designed a personalized scaffold fixation device by using the technology features of 3D printing. The scaffolds were firstly divided into different areas according to the different roles of different parts of the femoral scaffold, and then, the appropriate porous structures were selected for modular repair according to the functions of the corresponding areas. For the fixation of femur scaffold, according to the technical characteristics of 3D printing, the integrated fixation device with anatomical shape matching femur is designed, which can be well matched with the host bone tissue. The mechanical properties of homogeneous porous scaffolds and stiffness gradient scaffolds were analyzed by finite element analysis. On this basis, the displacements of the bone and the scaffold in different fixation modes were analyzed, and the stress and strain of the fixation device and the scaffold were compared.

## Materials and methods

### Establishment of 3D model of femur

**Fig. 1:**
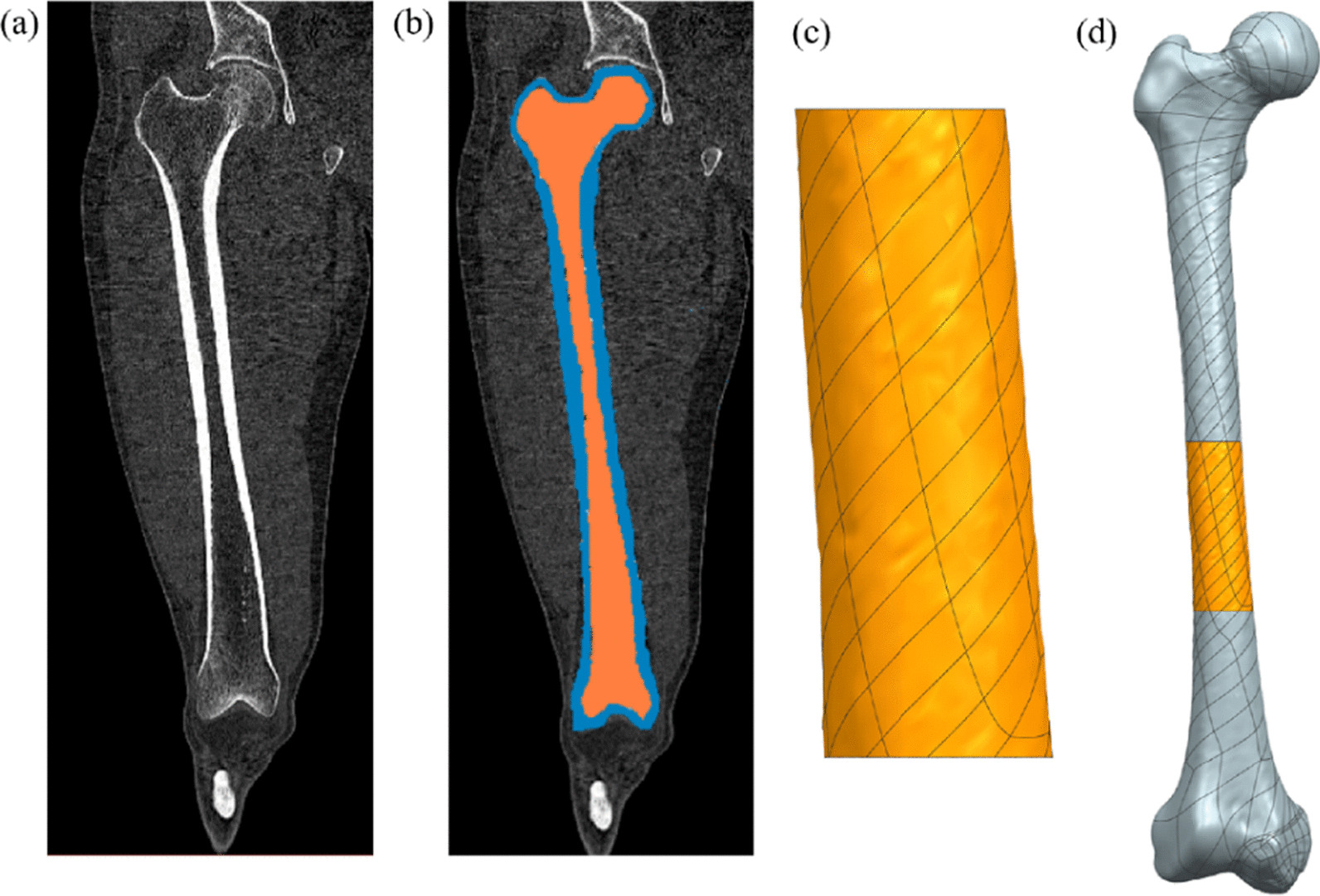
Establishment of 3D model femur. **a** Comparison of gray values between different tissues in human body; **b** threshold segmentation is performed for different tissues; **c** 3D model of the bone defect obtained by mirroring; **d** matching of 3D model of the defect with the host bone tissue

For the reconstruction of the 3D model of femur, CT data of the patient's femur should be scanned first, and DICOM3.0 format images are collected from the CT equipment. For the femoral CT data, we adopted the CT data used in the previous study and analyzed the healthy femoral CT data of a 40-year-old male volunteer (75 kg) [25]. Male volunteer hip tomography data obtained from CT scans were imported into Mimics 21.0 (Materialise's Interactive Medical Image Control System, Materialise, Belgium), and different gray values of different human tissues were utilized for threshold segmentation of CT data, as shown in Fig. 1a, b. Due to the non-uniform characteristics of human tissue itself, the 3D bone tissue model extracted from Mimics will have certain defects (such as holes and burr), which need to be repaired during biomechanical analysis to obtain the bone tissue structure of normal human anatomy. In this paper, Magics 22.0 (Materialise, Belgium) software was used to repair these defects, resulting in a 3D model of femur in Fig. 1d.

### Functional division of femoral scaffold

3D printing technology can preparation personalized femur fixation devices to match human bone tissue to achieve a perfect fit. Generally, because the bone tissue at the osteotomy site has been diseased, personalized design cannot be carried out according to the anatomical shape of the diseased bone tissue. It was assumed that the left and right femurs of the human body were symmetrically distributed. A 3D model of the healthy femur at the same position on the opposite side was first established, and then, the 3D model of the femur defect after tumor resection was obtained by mirror image. Figure 1c shows the 3D model of the mirrored femur defect site. In this way, the anatomical model of the defect site can basically completely match the host bone tissue after tumor resection, as shown in Fig. 1d. According to the study of the length of femur osteotomy by Benevenia et al. [26], the average length of femur osteotomy in general patients is 90 mm, so the length of femur intercepted in this design is 90 mm.

**Fig. 2:**
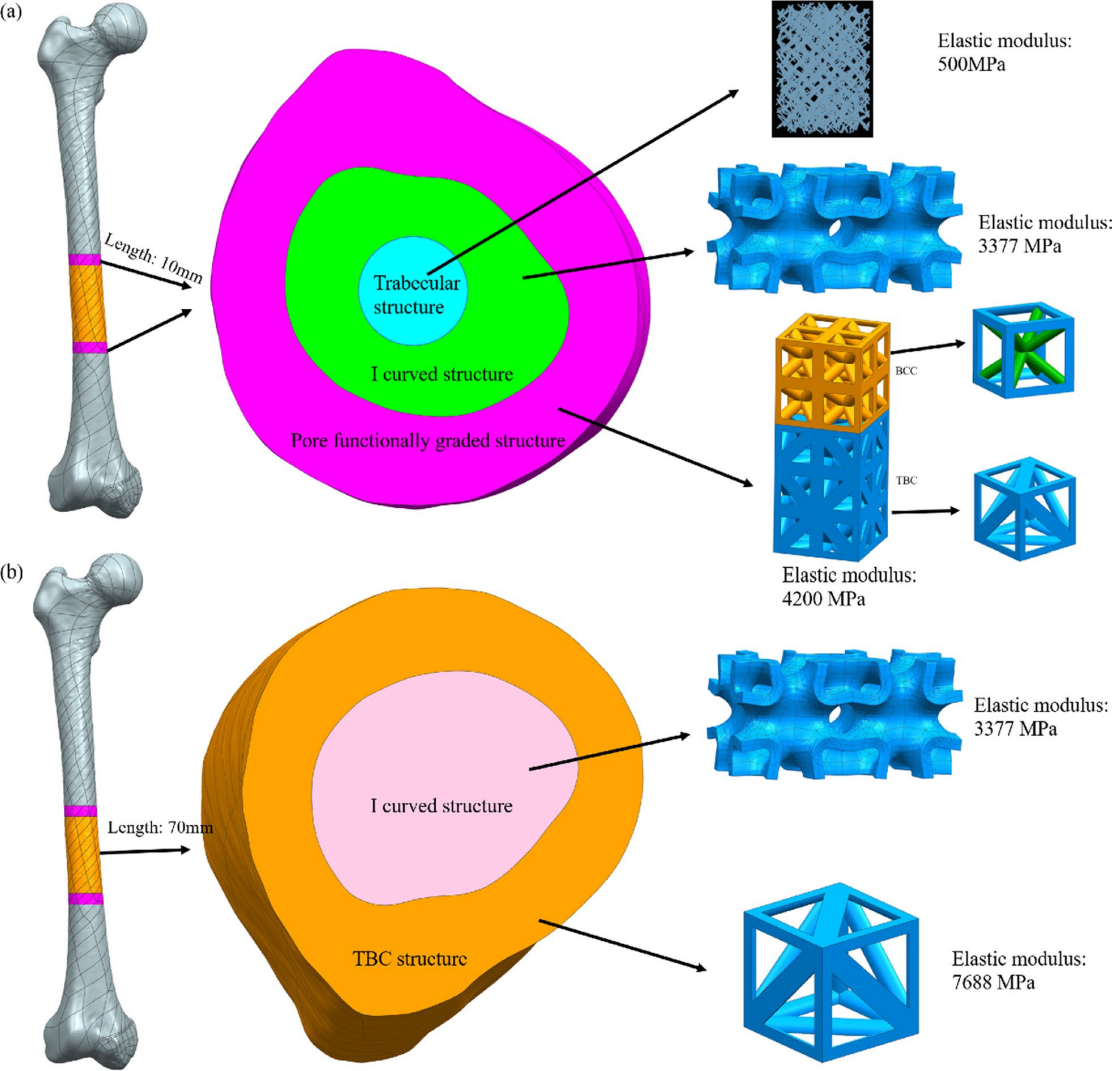
Schematic diagram of modular division of porous scaffold. **a** Interface module; **b** load-bearing module

3D-printed porous titanium alloy scaffolds need to have good biological properties to induce bone tissue into the interior, forming long-term biological fixation [27]. At the same time, for the large segment of femur defect, the scaffold also plays a mechanical support role to bear the load of the daily activities of femur, but the mechanical and biological properties of the scaffold are not optimal at the same time. According to the characteristics of the femur scaffold, the scaffold was divided into interface module and load-bearing module along the axis, as shown in Fig. 2. The interface module needs to promote the good growth of host bone tissue into the scaffold to form biological fixation [28], so it mainly plays a role in inducing the growth of bone tissue. As for the load-bearing module, it is difficult for the bone tissue to grow in the distance from the bone tissue [29], so it mainly plays the role of bearing the load of human daily activities. The length of the upper and lower interface modules is 10 mm, and the length of the load-bearing module is 70 mm, as shown in Fig. 2.

3D-printed porous titanium alloy has good mechanical and biological properties. We used pore functionally graded porous titanium alloy composed of body centered cube (BCC, porosity 55%) and tetrahedral structure (TBC, porosity 65%) to repair the femur defect [30]. For the cancellous bone part, we used the research results of Wang et al. [5] and used the imitation of trabecular structure with 86% porosity to repair. Our previous studies showed that I structure was also suitable for repairing cancellous bone [31]. In order to avoid stress concentration, I structure was used for transition between cortical bone and cancellous bone during design, as shown in Fig. 2a. TBC structure and I structure were used to repair the loaded cortical bone and cancellous bone, respectively, as shown in Fig. 2b. BCC structure, TBC structure and I structure unit size are 2 mm, and trabecular structure can be automatically generated based on the desired size. The selection of porous materials in different parts is shown in Table 1. Existing studies have shown that 3D printing technology can be used to prepare pore gradient scaffolds [6, 30]. The porous structure selected in this design has been prepared by 3D printing technology and has good mechanical and biological properties, so the designed femoral scaffolds can be prepared by 3D printing technology.

**Table 1:** Gradient design of femur scaffold structure and mechanical properties of each module [5, 30, 31]

Main module	Submodule	Porous structure	Elastic modulus/MPa	Poisson's ratio
Interface module	Interface cortical bone module	Pore functionally graded structure (BCC + TBC)	4200	0.3
	Interface transition module	I structure with 65% porosity	3377	0.3
	Interface cancellous bone module	Imitation of trabecular structure with 86% porosity	500	0.3
Load-bearing module	Load-bearing cortical bone modules	TBC structure with 55% porosity	7688	0.3
	Load-bearing cancellous bone module	I structure with 65% porosity	3377	0.3

### Design of femur scaffold fixation device

**Fig. 3:**
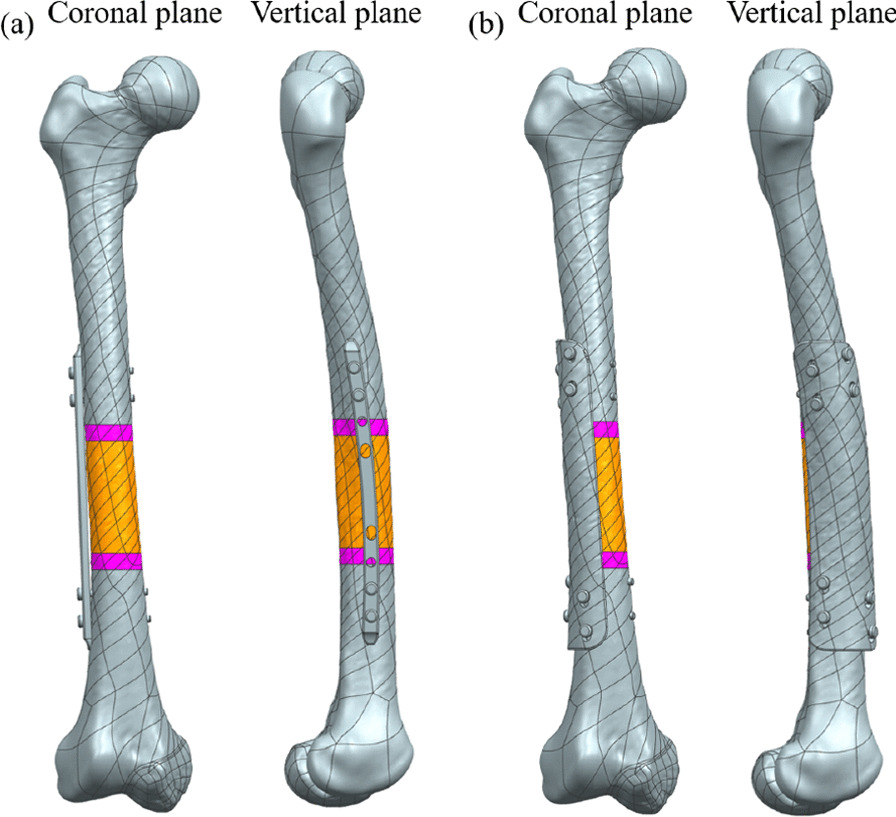
Fixation device design. **a** Plate fixation; **b** integrated fixation

The traditional femur scaffold is fixed by steel plate and fixation screw, which cannot completely fit the bone tissue, as shown in Fig. 3a. In order to make the implant fit well with the host bone tissue, the integrated fixation device is designed according to the anatomical shape of the patient's defect site. Generally, the surgical approach for femur implantation is lateral to the femur, and the muscle opening angle during the operation is about 100°. The integrated fixation device sets two rows of threaded holes in the lateral and anterior sides of the femur at an angle of 60°, and leaked half of the femur anteriorly for intraoperative observation. This personalized and integrated design of bone repair scaffold reduces the fixation time between traditional prostheses and fixation devices, thus reducing the surgical time. The upper and lower parts of the integrated fixation device are 50 mm, and 4.5 mm tapping screws are used for fixing. After the osteotomy site and fixed length were determined, the normal femur was cut out on the cutting surface above and below the fixation device and scaled up to 1.3 times. After the enlarged femur is assembled back to the original site, the normal femur is subtracted from the enlarged femur and the excess part is removed, leaving only 100°. In this way, a fixation device that completely matches the host bone tissue can be obtained, as shown in Fig. 3b.

### Analysis of the stability of femur scaffold

#### Boundary and loading conditions

**Fig. 4:**
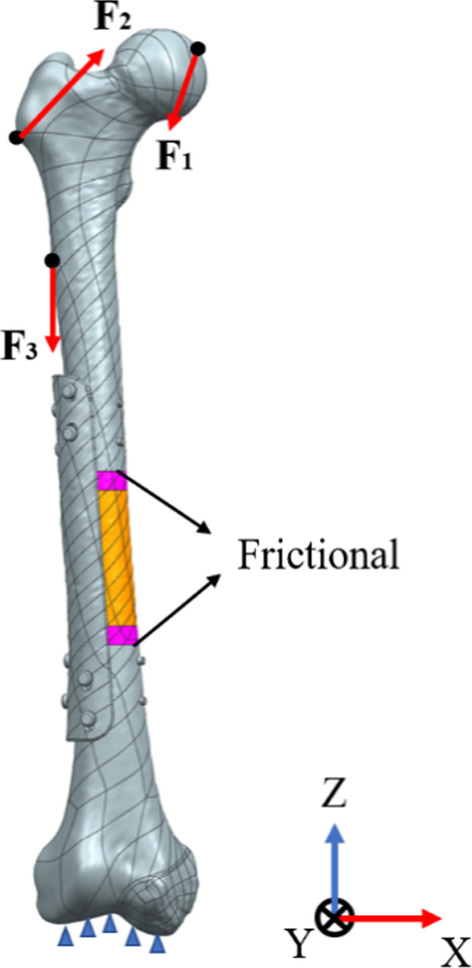
Boundary and loading conditions

ANSYS18.2 (Swanson Analysis, Canonsburg, PA, USA) software was used to analyze the stress and strain of different scaffolds and the relative displacement between bone and scaffold. Bergmann et al. [32] studied the daily activity load data of human daily activities, and this paper used the research results to load the model. The hip force is applied to the center of the femur head, and the muscle and ligament force is applied to the femur. The contact between cortical bone and cancellous bone was also set as binding constraint, the distal femur was completely fixed constraint, and the friction constraint was used between the scaffold and host bone with a friction coefficient of 0.4, as shown in Fig. 4. Due to the irregular structure of femur, the mesh in finite element is divided by tetrahedral mesh element, and the element type is solid element. Table 2 shows the values of each load force in each direction of the coordinate system.

**Table 2:** The magnitude of loading forces in all directions in femur finite element simulation

Load case	*X* direction (% body weigh)	*Y* direction (% body weigh)	*Z* direction (% body weigh)
*F*_1_	− 54	32.8	− 229.2
*F*_2_	64.7	− 15.2	80.7
*F*_3_	− 0.9	− 18.5	− 92.9

#### Stress analysis

Bone is a mechanical sensing organ, and studies have shown that the stress and strain of bone tissue have an important effect on bone regeneration [33]. In order to explore the stress and strain of homogeneous scaffolds and stiffness gradient scaffold on the host bone, especially the contact parts of the scaffolds, we analyzed the stress and strain of homogeneous scaffolds and stiffness gradient scaffold under the condition of daily activity load of human body under the condition of integrated fixation. The homogeneous scaffold adopts TBC structure with porosity of 55% (elastic modulus: 7688 MPa, Poisson's ratio: 0.3). The material properties of the stiffness gradient scaffold are shown in Table 1. We assume that all materials are homogeneous, isotropic and linearly elastic. The elastic modulus of cortical bone and cancellous bone was 1700 MPa and 500 MPa, respectively, and Poisson's ratio was 0.3 [25, 34].

#### Displacement analysis

**Fig. 5:**
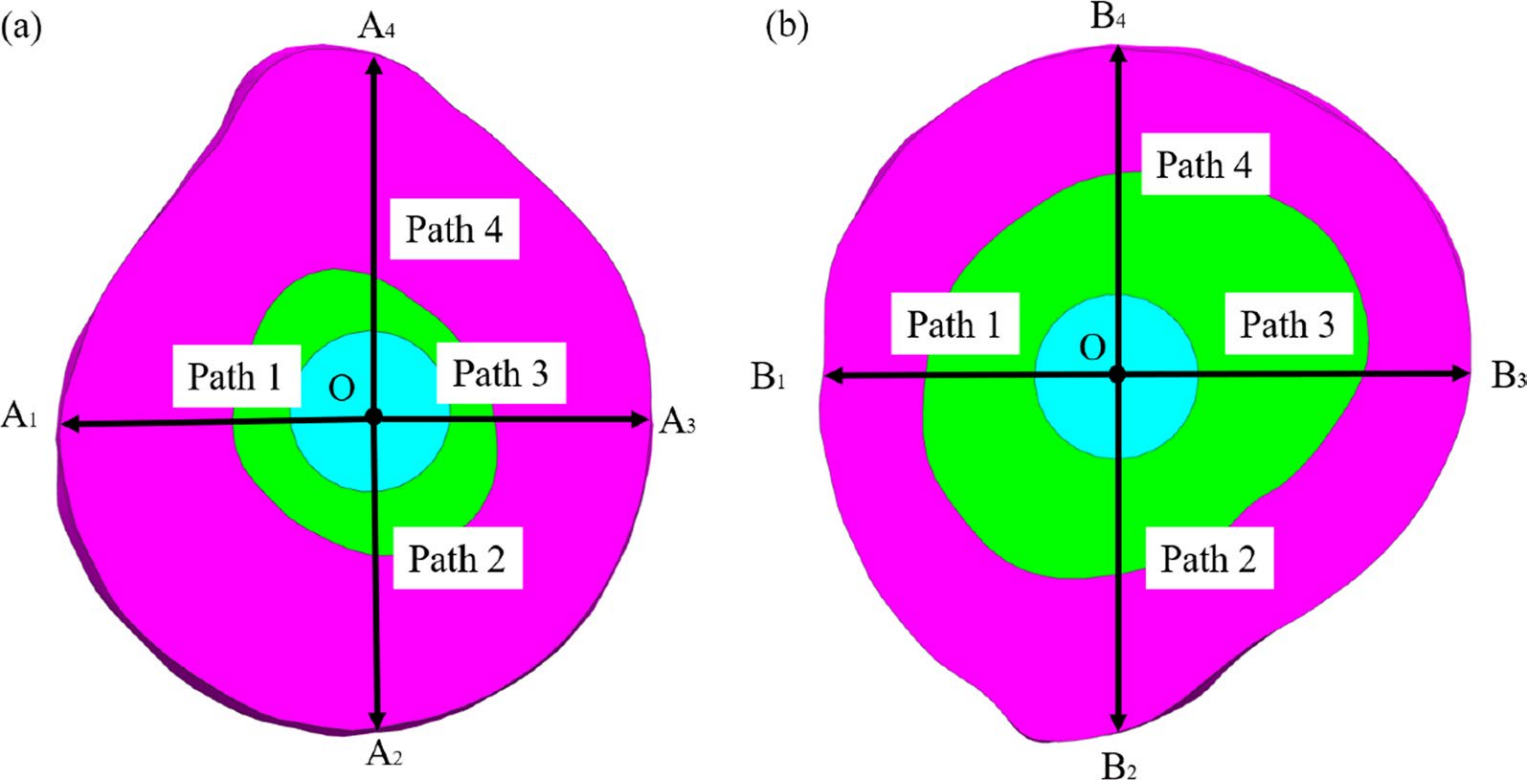
Four paths of the bone-scaffold interface. **a** Proximal femur; **b** distal femur

Studies have shown that when the displacement between bone and scaffold exceeds the critical value (150 µm), it will promote the growth of fibrous hoof tissue and inhibit the growth of bone tissue [35]. Therefore, the displacement between bone tissue and scaffold was used to evaluate the stability of the fixation method. Four different paths at the bone-scaffold interface were taken for analysis, and the displacements of host bone and porous scaffold on the four paths were calculated by using finite element analysis, respectively, and then, the relative displacements of the two were calculated. Figure 5 shows a schematic of the four selected paths.

## Result

In order to ensure the accuracy of the calculation results, the convergence analysis of the grid was carried out. The number of nodes in the model is about 350,000, and the number of grids is about 190,000. In order to solve the convergence of the calculation results, the number of model nodes is doubled, and a fine grid model is established. The peak displacement difference is less than 5%. The results show that the grid model is convergent.

**Fig. 6:**
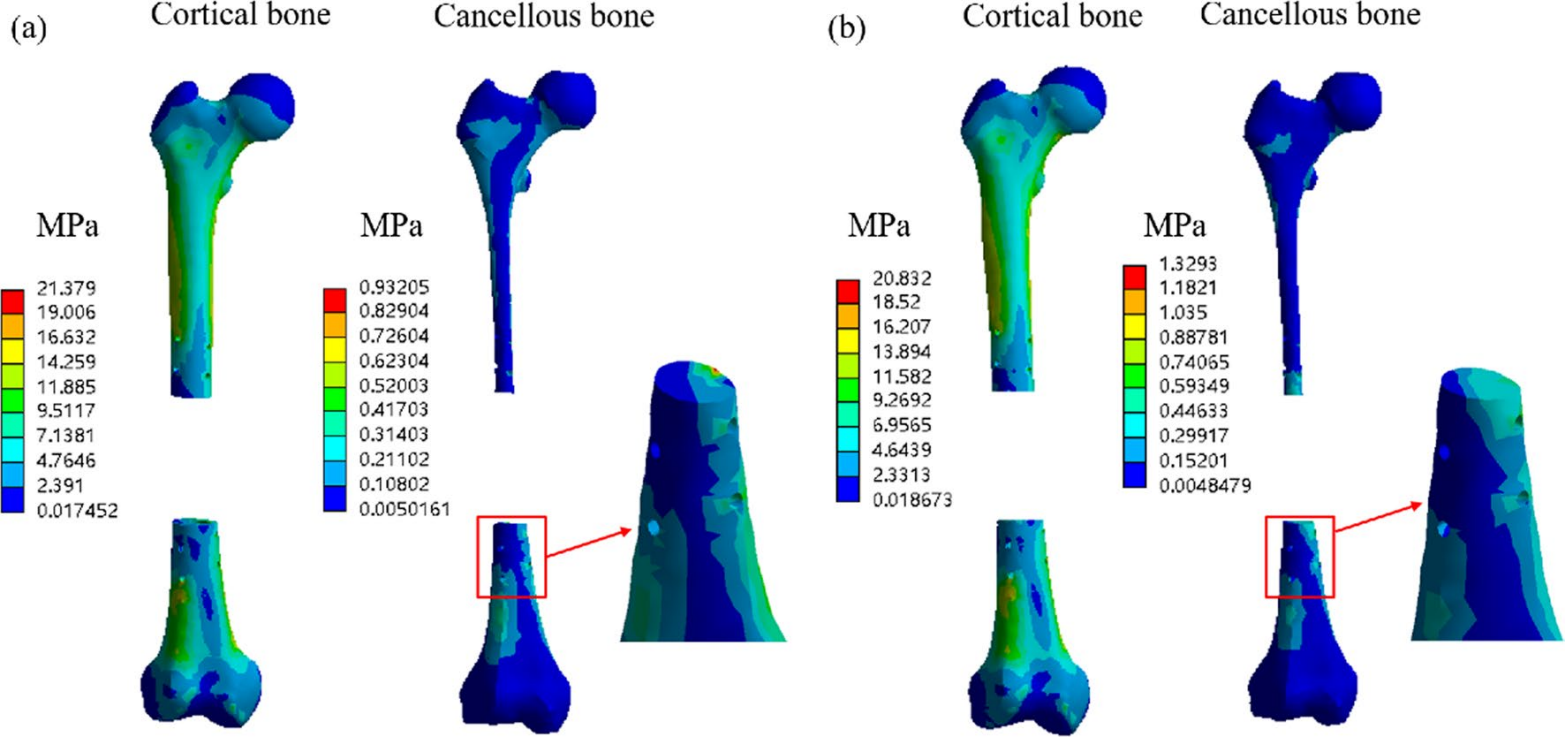
Bone stress cloud of homogeneous scaffolds and modular scaffolds. **a** Homogeneous; **b** stiffness gradient

Figure 6 shows the stress and strain clouds of homogeneous scaffolds and modular scaffolds under the condition of integrated fixation. In order to see the stress and strain of cancellous bone more clearly, we enlarged the cancellous cloud image around the bone-scaffold interface. Considering that the proximal and distal femur interfaces are basically the same, only the stress and strain cloud image of the distal femur is included here. It can be seen from the figure that there was no significant difference in the stress of cortical bone around the two-fixation device, indicating that the introduction of interface module did not change the overall stress of bone tissue. However, for cancellous bone, the stress of the host bone in the homogeneous scaffold group was less than that in the modular scaffold group. Moreover, it can be seen from the enlarged figure that the stress of the cancellous bone caused by the homogeneous scaffold was not uniform, and there was stress concentration near the cortical bone.

**Fig. 7:**
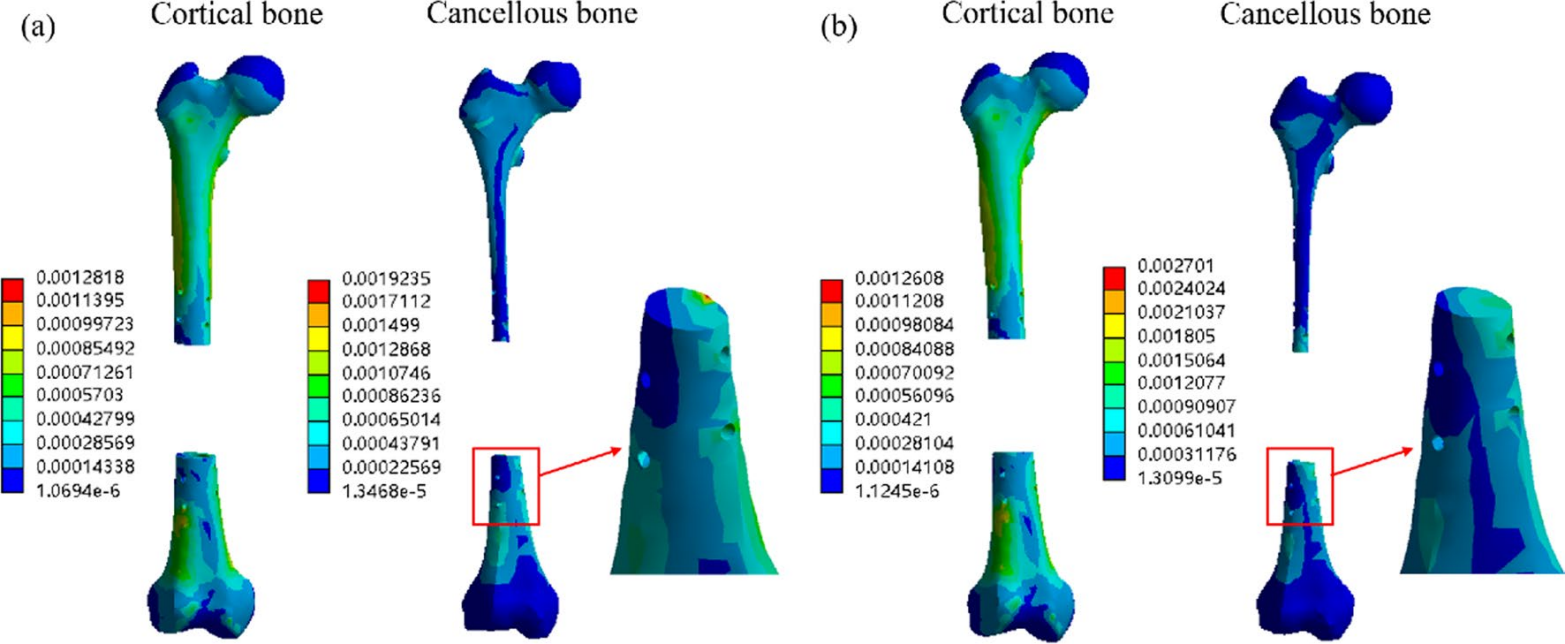
Cloud image of bone strain of homogeneous scaffolds and modular scaffolds. **a** Homogeneous; **b** stiffness gradient

Similar to the stress, the strain on cortical bone of homogeneous scaffold and modular scaffold was basically the same. However, for cancellous bone, there is a large gap between the two group, and the strain of cancellous bone with homogeneous porous scaffolds is small and unevenly distributed, as shown in Fig. 7. Therefore, modular scaffolds were used for further analysis.

**Fig. 8:**
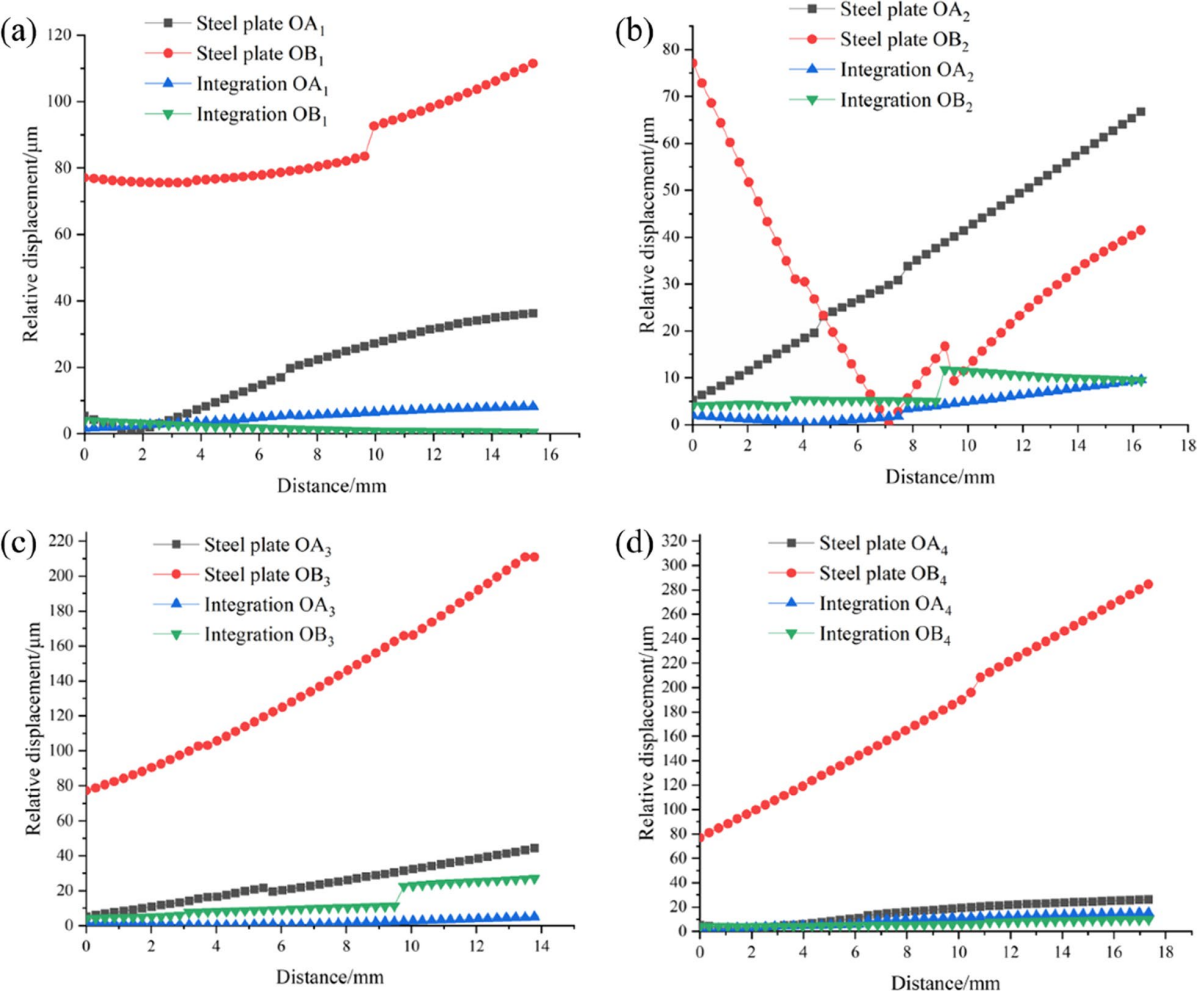
Relative displacements on four paths with different fixed modes. **a** Path1; **b** path 2; **c** path 3; **d** path 4

Figure 8 shows the relative displacement analysis of the scaffold and host bone in the four paths shown in Fig. 5 for traditional plate fixation and integrated fixation. As can be seen from the figure, the relative displacement peaks of integrated fixation are 8.2 μm, 11.8 μm, 27.1 μm and 15.4 μm, respectively, while the relative displacement peaks of steel plate fixation are 111.4 μm, 77 μm, 211 μm and 284.6 μm, respectively. The displacement of the integrated fixation method in four paths is smaller than that of the traditional steel plate fixation.

**Fig. 9:**
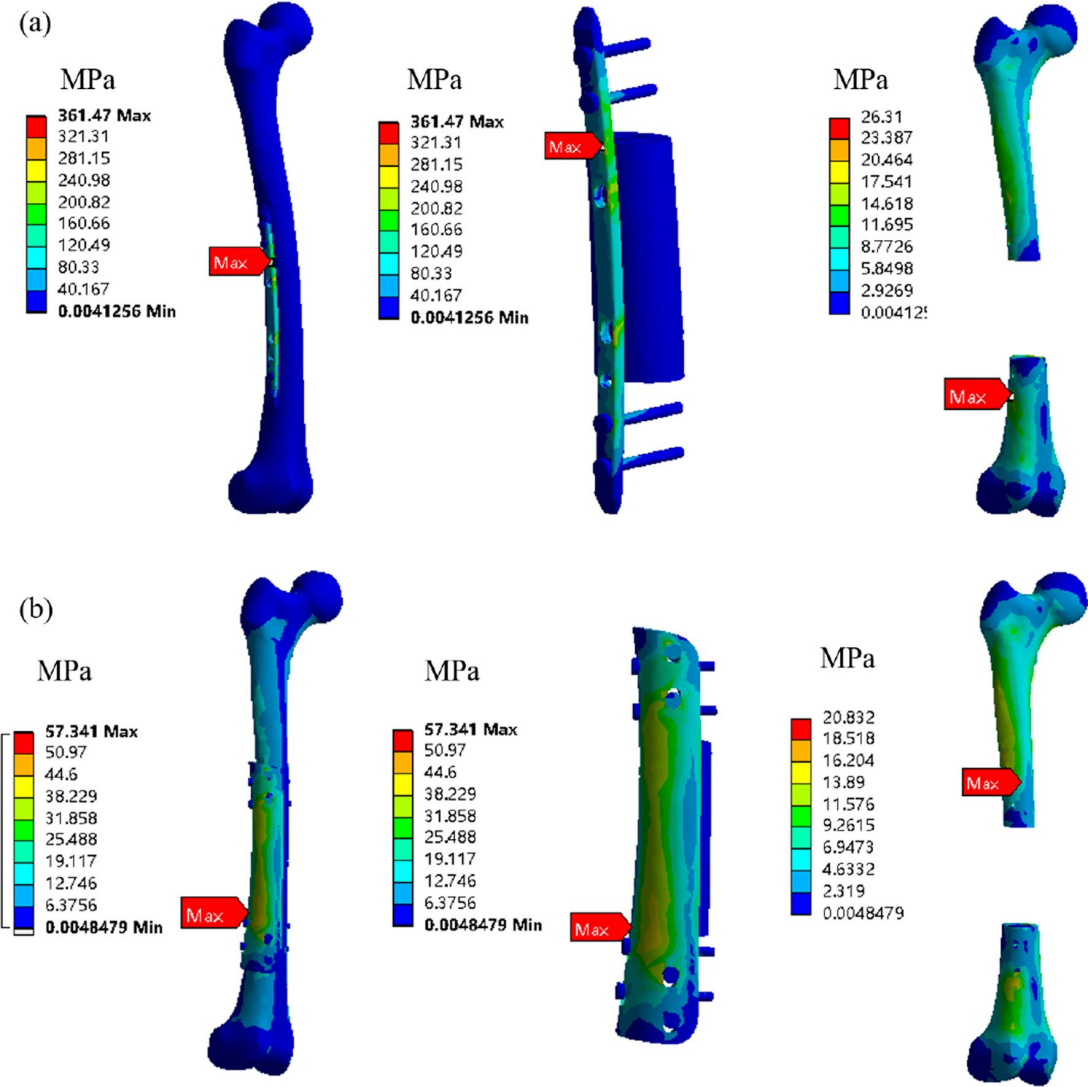
Stress cloud map of different fixation methods. **a** Plate fixation; **b** integrated fixation

Figure 9 shows the stress cloud of traditional plate fixation and integrated fixation. On the whole, the stress of integrated fixation is much less than that of steel plate fixation, and the stress concentration of steel plate fixation is obvious. Most of the stress is concentrated on steel plate, and the stress distribution of integrated fixation is more uniform. There is no significant difference between plate fixation and integrated fixation for host bone.

## Discussion

In this paper, in order to reduce the calculation time, we homogenize the porous structure and treat it as a uniform continuum. Ali Entezari et al. [36] showed in their study of porous femur scaffold that homogenized treatment method could obtain similar results as that of porous structure, so it is feasible to homogenize treatment of porous structure in this paper. Figures 6 and 7 show the stress and strain conditions of homogeneous scaffolds and stiffness gradient scaffolds under integrated fixation, respectively, and there is not much difference between them for cortical bone. This may be due to the fact that the cortical bone of the interface part of the modular scaffold adopts pore functionally graded structure, and the elastic modulus is 4200 MPa, which is not much different from the homogeneous scaffold 7688 MPa. However, in the cancellous bone part, there is a large gap between the homogeneous scaffold and the stiffness gradient scaffold in terms of stress and strain, because the elastic modulus of the homogeneous scaffold in the cancellous bone part is still 7688 MPa, while the stiffness gradient scaffold uses the imitation of trabecular structure to match the mechanical properties of the cancellous bone part. Studies have shown aseptic loosening of bone implants in vivo, which is caused by the lack of good biological fixation between the host bone and the implant [37, 38]. The growth of bone tissue has a great relationship with the mechanical stimulation, and the size of mechanical stimulation in a certain range determines the depth of bone ingrowth [6, 39, 40]. Stiffness gradient scaffolds have low elastic modulus in the cancellous bone to avoid the stress shielding, so the cancellous bone can also be stimulated by mechanical stimulation to promote the growth of bone tissue.

After the design of porous scaffolds, the fixation mode has an important effect on the stability of scaffolds after implantation. The higher the stability, the smaller the relative displacement between the scaffold and the host bone, and more conducive to the growth of bone tissue. It can be seen from Fig. 8 that the displacement of the plate fixation method on Path 3 and Path 4 exceeds 150 μm, which does not meet the stability requirements. As can be seen from the two design diagrams in Fig. 3, the traditional plate fixation screws are almost in the same straight line, and the plate only contacts the femur at the upper and lower ends and does not contact the bone tissue in the middle part. In Fig. 8b, the displacement of Path 2 during plate fixation in OB_2_ first decreased and then gradually increased, which may be due to the torsional force exerted on the distal femur, and the screws fixed by plate fixation almost in a straight line could not properly fix the torsional force. The integrated fixation method is personalized designed according to the femur of each patient and obtained by means of Boolean operation, which can perfectly match the anatomical shape of femur. In addition, the integrated fixation device and porous scaffold can be printed together by 3D printing, which avoids the displacement between the fixation device and porous scaffold and makes the operation more convenient.

After implantation, the femur scaffold needs to bear the fatigue load of human daily activities, so its stress condition also has a crucial influence on the long-term stability of the scaffold. Figure 9 shows the stress cloud diagram of plate fixation and integrated fixation, from which it can be seen that the stress of plate fixation is much higher than that of integrated fixation. For the finite element analysis of human femur stress, the stress on the femur is mainly distributed in the distal end of the femur, while the stress on the proximal end of the femur is small, which is consistent with the research results of Wang et al. [25]. In comparison with the work of Hazlehurst et al. [41], a similar trend was observed in this paper, with stress values within the same order of magnitude. The maximum stress of both plate fixation and integrated fixation occurs at the threaded hole, but the maximum stress of plate fixation (361 MPa) is 6.3 times that of integrated fixation (57 MPa). This phenomenon may be caused by the fact that the plate fixation is a straight line, which tends to produce displacement between the bone and the scaffold interface when subjected to bending and torsional forces, resulting in stress concentration. On the contrary, the integrated fixation has a certain angle around the femur, and the fixation screw can also play a good role in fixing the bending and torsional forces in different directions of the femur. Huang et al. [42] evaluated the therapeutic efficacy of personalized inserted femur prostheses, and the research results showed that customized modular femur prostheses had good clinical effects, and the implantation of artificial metal femur repair scaffolds could quickly restore the function of the defect site and had good initial stability. However, the stress shielding effect caused by the huge difference in stiffness between pure metal scaffolders and host bone is one of the main reasons for the failure of femoral implants [43]. The long-term effectiveness of stents has been the primary goal of scaffold design [44]. Meanwhile, the traditional pure metal femur prosthetics are manufactured by mechanical processing, and the anatomical shape is difficult to match the shape of human bone defect, which will inevitably lead to the change of the force line of human lower limbs. The integrated fixation and porous scaffolds are prepared by 3D printing without displacement, and it fits perfectly with the host bone at both ends. Compared with the linear fixation of the steel plate, the integrated fixation is a surface fixation, and the force it receives is evenly distributed on the whole fixation device, so the stress concentration phenomenon is small. Moreover, the plate fixation has only 4 screws in a straight line, while the integrated fixation has 8 screws in different directions, which only has a dispersive effect on the force received by the femur, and therefore, the stress is less. The yield strength of 3D-printed titanium alloy is about 980 MPa [45], and many existing studies also show that the normalized fatigue strength of 3D-printed porous structure is within the range of 0.1–0.25 [46]. From this point of view, the stress levels shown in Fig. 9 can meet the fatigue strength requirements. Moreover, as the bone tissue grows into the porous scaffold and forms a integrated with the scaffold, its stress characteristics will change, and its fatigue performance will be further improved [43].

Compared with traditional bone implants, integrated stiffness gradient scaffolds can design personalized implants and fixation devices according to different anatomical shapes of human femur [47], improving the stability of fixation. Compared with homogeneous porous scaffolds, the stiffness gradient scaffolds used the same elastic modulus structure as the homogeneous scaffolds in the bearing part and reduced the elastic modulus of the scaffolds at the bone-scaffold interface. The stiffness gradient scaffolds were used to repair cortical bone and cancellous bone with different structures. This design method can increase the structural strain of the bone-scaffold interface and improve the biological activity of the scaffold. At the same time, the design of stiffness gradient is conducive to the growth of bone tissue, so it is a better treatment scheme for bone defects.

## Conclusion

This paper proposes a stiffness gradient scaffold design method, which divides the scaffold into different modules according to the different roles of different parts of the scaffold. Then, according to the existing research, different porous structures were used to repair the corresponding modules. Finally, an integrated fixation device was designed according to the characteristics of femur surgery. Two kinds of scaffolds and two kinds of fixation methods were analyzed by finite element method. The results show that the stiffness gradient scaffold can generate greater strain than the homogeneous scaffold, and the integrated fixation method has better stability and less stress than the plate fixation. Therefore, we believe that stiffness gradient scaffold design combined with integrated fixation devices can be used to repair large femur defects.

Different from other studies, this study proposed to divide the bone scaffold into bone-scaffold interface region and bearing region, and to use matching porous structures for repair according to the different roles of different regions. The shortcoming of this study is that it did not use 3D printing technology to prepare scaffold samples and implant the samples into the femur for mechanical experiments. Another shortcoming of this study is that animal experiments were not used to verify the biological activity of the stiffness gradient scaffold. We will continue to conduct further research in the future work.

## Data Availability

The datasets used and/or analyzed during the current study are available from the corresponding author on reasonable request. All data generated or analyzed during this study are included in this published article.

## Abbreviations

CT: Computed tomography
3D: Three-dimensional
BCC: Body centered cube
TBC: Tetrahedral structure
